# Involuntary Eye Movements as a Clue to Diagnosis

**DOI:** 10.1002/mdc3.70233

**Published:** 2025-07-14

**Authors:** Victor Rebelo Procaci, Raphael Pinheiro Camurugy da Hora, Thiago Yoshinaga Tonholo Silva, Orlando Graziani Povoas Barsottini, José Luiz Pedroso

**Affiliations:** ^1^ Department of Neurology, Ataxia Unit Universidade Federal de São Paulo São Paulo Brazil

**Keywords:** movement disorder, acetazolamide, FGF14, SCA27A, ataxia

## Question

A 15‐month‐old boy was referred for evaluation due to involuntary eye movements (Video 1) and episodes of truncal and gait ataxia triggered by fever. During these episodes, the patient experienced difficulty assuming a sitting position, and gait was more unstable with frequent falls, lasting from minutes to an hour. Some episodes consisted solely of truncal ataxia. There was no history of antidopaminergic medication. The patient was born healthy after an uncomplicated, full‐term pregnancy to nonconsanguineous parents and exhibited no developmental delays. Brain magnetic resonance imaging (MRI) was normal, and an electroencephalography (EEG) during those episodes showed no seizures.

**Video 1:** Part 1 and Part 2: involuntary eye movements characterized by episodes of tonic upgaze deviation triggered by fever. The patient is seen playing and interacting during the video. Part 3: episode of truncal ataxia and difficulty maintaining an upright position. Part 4: the patient is able to stand and walk, with slight difficulty in gait between attacks.

## What Is the Phenomenology Presented in the Video?

1. Vertical nystagmus
2. Opsoclonus
3. Paroxysmal tonic upgaze
4. Oculogyric crisis

## Answer

Paroxysmal tonic upgaze (PTU) in childhood was described in 1988, characterized by episodes of constant or variably sustained conjugate upward deviation of the eyes, downbeat saccades during attempted downgaze, normal horizontal eye movements, commonly associated with neck flexion. PTU has been described in genetic mutations in *CACNA1A*, *GRID2*, and others.^1^ Carbonic anhydrase inhibitors can be considered to manage PTU irrespective of the etiology.^2^

Oculogyric crisis (OGC) is an important differential of PTU. OGC is a form of dystonia, characterized by paroxysmal, tonic, and conjugate ocular deviations caused by sustained contraction of the extraocular muscles. Episodes last from minutes to hours, most commonly involving upward gaze deviation, and may be associated with other dystonic symptoms (Table 1). OGC can occur in genetic disorders that impair dopamine and as a complication of antidopaminergic medication.^1^

**TABLE 1:** Clinical characteristics of PTU and clinical criteria for OGC

PTU	OGC
**Clinical characteristics**	**Required criteria**
Onset usually under 1 yr of age* Episodes of variably sustained conjugate upward deviation of the eyes*, with neck flexion apparently compensating for the abnormal eye position Downbeating saccades in attempted downgaze* Normal horizontal eye movements* Diurnal fluctuation of symptoms* Frequent relief by sleep* Exacerbation with febrile illnesses* Varying degrees of ataxia* Neurological examination usually otherwise normal* Absence of deterioration during long‐term follow‐up* Eventual improvement* Usually negative investigations, including imaging*, EEG*, and CSF neurotransmitters	Tonic, conjugate deviation of eyes Minutes to hours in duration Consciousness preserved
	**Supportive criteria**
	Preceded by anxiety, discomfort† Patient is aware of and bothered or disabled by the ocular deviations† Associated dystonia† Associated with a low dopamine state† Improved by anticholinergics or dopaminergic medication†

Abbreviations: PTU, paroxysmal tonic upgaze; OGC, oculogyric crisis; EEG, electroencephalogram; CSF, cerebrospinal fluid.

* PTU criteria present in our patient.

^†^ Supportive criteria for OGC not present in our patient.

Exome sequencing was ordered under the suspicion of episodic ataxia with symptoms triggered by fever, and a de novo heterozygous variant of uncertain significance (VUS) was identified in NM_004115.4(FGF14):c.353G>T(p.Gly118Val). Although the genetic testing revealed a VUS, symptoms were suggestive of episodic ataxia, which could be a manifestation of spinocerebellar ataxia type 27A. Further analysis of this variant is warranted. The patient was treated with acetazolamide and had improvement of the episodic symptoms.

## Author Roles

(1) Research project: A. Conception, B. Organization, C. Execution; (2) Statistical analysis: A. Design, B. Execution, C. Review and critique; (3) Manuscript: A. Writing of the first draft, B. Review and critique.

V.R. P.: 1A, 1B, 1C, 3A

R.P.C. da H.: 1B, 3B

T.Y.T.S.: 1B, 3B

O.G.P.B.: 3B

J.L.P.: 1A, 1B, 3B

## Disclosures

**Ethical compliance statement**: This study was approved by our local ethics institution. Patient consent form was obtained. We confirm that we have read the journal's position on issues involved in ethical publication and affirm that this work is consistent with those guidelines.

**Funding Sources and Conflict of Interest**: This researh was funded by the Coordenação de Aperfeiçoamento de Pessoal de Nível Superior (CAPES), which is the Brazilian Federal Agency for Support and Evaluation of Graduate Education—a foundation linked to the Ministry of Education that supports research in Brazil. The authors declare that there are no conflicts of interest relevant to this work.

**Financial disclosures for the previous 12 months**: The authors declare that there are no additional disclosures to report.

## Data Availability

Data sharing not applicable to this article as no datasets were generated or analysed during the current study.
